# Hypercholesterolemia and related risk factors in a cohort of patients with diabetes and hypertension in Maputo, Mozambique

**DOI:** 10.11604/pamj.2021.38.102.27284

**Published:** 2021-01-29

**Authors:** Fausto Ciccacci, Noorjehan Majid, Sandro Petrolati, Mustafa Agy, Cacilda Massango, Stefano Orlando, Giovanni Guidotti, Paola Scarcella, Maria Cristina Marazzi

**Affiliations:** 1 UniCamillus, Saint Camillus International University of Health Sciences, Rome, Italy,; 2 Disease Relief through Excellence and Advanced Means (DREAM) Program, Community of Sant'Egidio, Maputo, Mozambique,; 3 Department of Cardioscience, San Camillo Hospital, Rome, Italy,; 4 Department of Biomedicine and Prevention, University of Rome Tor Vergata, Rome, Italy,; 5 Azienda Sanitaria Locale (ASL) Roma 1, Rome, Italy,; 6 Libera Università Maria Santissima Assunta (LUMSA), Rome, Italy

**Keywords:** Hypercholesterolemia, non-communicable diseases, cardiovascular risk factor

## Abstract

**Introduction:**

some studies reported that 25.5% of African population presents hypercholesterolemia; however, epidemiology of hypercholesterolemia in Africa is poorly described. Mozambique is experiencing a constant growth of non-communicable diseases, but scarce data are available about hypercholesterolemia. Our study aims at describing the prevalence of hypercholesterolemia in patients with diabetes and hypertension in Mozambique and investigate possible risk factors.

**Methods:**

we conducted a cross-sectional study involving all the patients diagnosed with hypertension and/or diabetes from June 2018 to November 2020 in the Zimpeto DREAM Centre (Maputo, Mozambique). For each patient, anthropometric, clinical and laboratory data were collected. Hypercholesterolemia was defined as total blood cholesterol >200 mg/dL. Univariable and multivariable analysis were perfumed.

**Results:**

a total of 885 patients were included, 76.2% (n=674) female. Hypertension alone was diagnosed in 670 (75.7%) patients, diabetes in 109 (12.3%) patients and 106 (11.9%) both diseases. Hypercholesterolemia was present in 410 (46.3%) patients and it was more prevalent in patients diagnosed with both diabetes and hypertension (52.8%), as compared to the patients diagnosed with hypertension (46.9%) or diabetes alone (36.7%). In the multivariable analysis, the only factors independently associated with hypercholesterolemia were female sex (aOR 1.77, 95% CI 1.26-2.48, p=0.001) and a body mass index >25kg/m^2^ (aOR 1.50, 95% CI 1.11-2.04, p=0.008).

**Conclusion:**

our results highlight the need for a specific focus on female and obese/overweight patients, especially if diagnosed with both hypertension and diabetes, to promptly detect metabolic disorders and establish temporary preventive measures for cardiovascular events.

## Introduction

According to the World Health Organization (WHO), a third of the global burden of ischemic heart disease is attributable to hypercholesterolemia, that represents a fundamental challenge for global health [1]. Prevalence of hypercholesterolemiain African countries is poorly described. According to some authors, the general prevalence in Africa is 25.5% [2]. In many low- and middle-income countries, surveillance and research on non-communicable diseases are still lacking [3,4]. However, the number of NCDs-related deaths is dramatically rising in the last decades [5,6]. Many observers claimed for action to control non-communicable diseases (NCDs) in Africa [7,8].

Mozambique is experiencing a constant growth of NCDs incidence and mortality in recent years, although infectious diseases are still burdening the country [9]. The prevalence of hypercholesterolemia is in line with that of the continent. Last national data available from WHO, report that prevalence of hypercholesterolemia (>5.0 mmol/L) in Mozambique was 25.2% in males and 24.9% in females in 2008 [10]. At the moment, insufficient data are available about the epidemiology of hypercholesterolemia in Mozambique. Our study aims at describing the prevalence of hypercholesterolemia in patients diagnosed with diabetes and hypertensionand investigate possible risk factors to identify specific risk groups.

## Methods

**Study design and setting:** we conducted a cross-sectional study on electronic medical records (EMR) of the Zimpeto DREAM Centre in Maputo, Mozambique. The Community of Sant´Egidio runs the Zimpeto Health Centre within the DREAM program and it was established in June 2018. The DREAM program is a public health program active in several African countries providing some health services: HIV care, TB treatment, HPV screening and treatment, hypertension and diabetes prevention and treatment [11-15].

**Study population:** we included all the patients diagnosed with hypertension and/or diabetes in the facility. In Zimpeto DREAM Centres all the patients are screened for diabetes and hypertension and if diagnosed, are followed up. In the present analysis we included all consecutive patients accessing the centre meeting the inclusion criteria. The inclusion criteria was: being diagnosed with diabetes and/or hypertension and undergo a full evaluation for non-communicable diseases from the beginning of the activities of the centre, up to the end of November 2020. Exclusion criteria were: age <18 years and pregnancy or breastfeeding.

**Data collection and definitions:** for each patient, anthropometric, clinical and laboratory data were collected. Hypertension and diabetes were diagnosed according to national guidelines. The better reference level for waist circumference (WC) in African population is under debate [16,17]. In absence of clear evidences, we followed the international standards, and we defined increased WC as WC >94cm in men and WC >80cm in women [18]. Hypertension was defined as blood pressure (BP) higher than 140 mmHg or 90 mmHg at three measurements [19]; diabetes was diagnosed if fasting blood glucose was higher than 126 mg/dL [20]. Hypercholesterolemia was defined as total blood cholesterol >200 mg/dL [1].

**Statistical analysis:** statistical data analysis was performed using SPSS 21 software (IBM Corp., Armonk, NY). Variables are presented as median with interquartile range (IQR). Crude and adjusted odds ratios (cOR and aOR) are reported with 95% confidence interval (CI). Chi-square test was used for the univariable analysis of factors associated with hypercholesterolemia. We included in the multivariable logistic regression all the variables that resulted in being associated with hypercholesterolemia at the univariable analysisat a 0.2 significance threshold.

**Ethical considerations:** all the services in the site of the study are delivered in collaboration and agreement with the local and national health authorities. The study used only clinical routine data collected during the service delivery andall the data were anonymized before extraction. For these reasons the study was exempted from specific consent.

## Results

**Figure 1:**
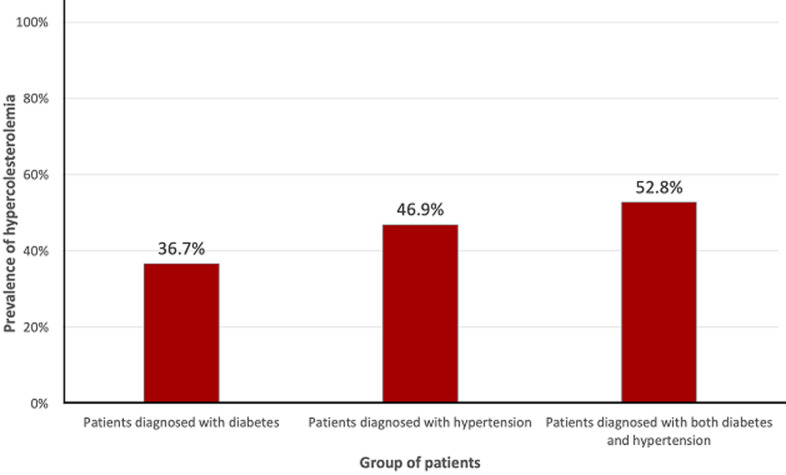
prevalence of hypercholesterolemia in patients with hypertension, diabetes, or both

On the whole, 885 patients were included; 76.2% (n=674) were female. The characteristics of the cohort are shown in Table 1. Hypertension alone was diagnosed in 670 (75.7%) patients, diabetes in 109 (12.3%) patients and 106 (11.9%) patients were diagnosed with both hypertension and diabetes. Among the 885 patients enrolled, 410 (46.3%) patients had hypecholesterolemia. Figure 1 shows the rate of hypercholesterolemia among the patients diagnosed with diabetes, hypertension or both. Hypercholesterolemia was more prevalent in patients diagnosed with both diabetes and hypertension (52.8%), as compared to the patients diagnosed with hypertension (46.9%) or diabetes alone (36.7%).

**Table 1:** characteristics of the cohort

Gender, n° (%)	
Female	674 (76,2)
Male	211 (23,8)
Median age, years (IQR)	57 (49 - 65)
Median BMI kg/m2, median, (IQR)*	28,0 (23,6 - 32,1)
Median waist circumference, cm (IQR)**	92 (81 - 104)
Median fasting blood glucose, mg/dL (IQR)***	96 (85 - 126)
Median diastolic blood pressure, mmHg (IQR) +	97 (86 - 107)
Median systolic blood pressure, mmHg (IQR)	167 (148 - 185)
Median blood cholesterol, mg/dL (IQR)	197 (167 - 229)


* n=847; ** n=743; *** n=835

Table 2 shows the results of the univariable and multivariable analysis. In the univariable analysis, the factors significantly associated with hypercholesterolemia were female sex, a body mass index (BMI) >25kg/m^2^and being diagnosed with hypertension. However, in the multivariable analysis, only female sex (aOR 1.77, 95% CI 1.26-2.48, p=0.001) and BMI >25kg/m^2^ (aOR 1.50, 95% CI 1.11-2.04, p=0.008) were independently associated with increased hypercholesterolemia.

**Table 2:** factors associated with hypercholesterolemia in univariable and multivariable analysis

		Normal cholesterol (<200 mg/dL)	Elevated cholesterol (>200 mg/dL)	Crude odds ratio (95% CI)	Adjusted odds ratio (95% CI)
Sex	M, % (n)	66.8% (141)	33.2% (70)	1.5 (1.48 - 2.83) p<0.001	1.7 (1.26 - 2.48) p=0.001
	F, % (n)	49.6% (334)	50.4% (340)		
Age	Adult, % (n)	54.4% (349)	45.6% (293)	1.1 (0.82 - 1.48) p=0.55	
	Elderly, % (n)	51.9% (126)	48.1% (117)		
BMI	BMI <25 kg/m2, % (n)	62.5% (173)	37.5% (104)	1.7 (1.27 - 2.29)	1.5 (1.11 - 2.04)
	BMI >25 kg/m2, % (n)	49.3% (281)	50.7% (289)	p<0.001	p=0.008
Waist circumference	Normal WC % (n)	56.4% (126)	43.6% (95)	1.2 (0.89 - 1.68) p=0.21	
	Increased WC, % (n)	51.4% (270)	48.6% (255)		
Diagnosis of hypertension	No hypertension, % (n)	63.3% (69)	36.7% (40)	1.5 (1.03 - 2.37)	0.7 (0.46 - 1.10)
	Hypertension, % (n)	52.3% (406)	47.7% (370)	p=0.03	p=0.12
Diagnosis of diabetes	No diabetes, % (n)	53.1% (356)	46.9% (314)	0.9 (0.67 - 1.24)	
	Diabetes, % (n)	55.5% (119)	44.7% (96)	p=0.57	

## Discussion

To our knowledge, our study is the first attempt to assess the prevalence of hypercholesterolemia in a population of patients accessing a health facility in Mozambique. In our cohort, 46.3% of patients presented hypercholesterolemia. It is worth to notice that these patients accessed the centre for other reasons and were subsequently diagnosed with hypertension and diabetes. Hypercholesterolemia was only a secondary diagnosis. The prevalence was even higher (52.8%) if considering patients diagnosed with both hypertension and diabetes. If other studies would confirm these data, a more systematic screening for hypercholesterolemia should be implemented in Mozambique for patients accessing health care for hypertension and/or diabetes.

The extensive review by Noubiap and colleagues involving 177 studies from several African countries (but not Mozambique), reported a pooled prevalence of hypercholesterolemia in patients with diabetes and hypertensionslightly lower than ours (respectively 38.0% and 34.4%). However, the trend is similar. Our results can highlight the hidden burden of dyslipidemia on patients accessing health care also in Mozambique. Blood cholesterol is not routinely assessed in many African countries and our results strengthen the advice for a full evaluation of patients even in African health facilities. Moreover, our data reported hypercholesterolemia as an independent condition, as it was independently associated only with female sex and increased BMI. Our cohort was disproportionally composed of women (76,2%). This finding is in line with the so called “gender paradox” and the lower rate of males seeking for care in comparison to women observed in many context [21,22].

The association with increased BMI was also described in other settings [23]. Interestingly, our results did not find any association between waist circumference and hypercholesterolemia. Although some studies found associations between central obesity and hypercholesterolemia, scholars are not unanimous about what is the best obesity index to correlate with metabolic disorders [23,24]. In our cohort, BMI was the only factor associated with hypercholesterolemia. The association with female sex is in line with data described in other African countries [25,26]. Similarly, the WHO reports that 11% of male and 24% of females are obese or overweight in Mozambique [6].

We are aware of some limitations of the present study. The absence of more detailed information about the cohort. Some critical information about other cardiovascular risk factors (such as familiarity, physical activity, tobacco and alcohol consumption) and lipidic asset (high-density lipoprotein (HDL), low-density lipoprotein (LDL) and triglyceride) were lacking. Notwithstanding these limitations, the main strength of our analysis is that it is based on field data, similar to the information that physician could access in everyday activity. In this perspective, our results strengthen the need for special attention to pay to particular groups of patients at higher risk for concomitant hypercholesterolemia.

## Conclusion

This study shows that about half of patients diagnosed with hypertension and diabetes had concomitant hypercholesterolemia. Independent factors associated with hypercholesterolemia included were female sex and being overweight. Consequently, our results suggest the need for a more specific focus on female and obese/overweight patients, especially if diagnosed with both hypertension and diabetes, to promptly detect metabolic disorders and establish temporary preventive measures for cardiovascular events.

### What is known about this topic

- Hypercholesterolemia is a prevalent health challenge in African countries burdening around 25.5% of the adult population;
- Hypercholesterolemia is strongly associated with other NCDs and mortality.

### What this study adds

- We found that a high proportion of patients diagnosed with diabetes and hypertension (46.3%) are concomitantly affected with hypercholesterolemia;
- Our study found that in Mozambican patients with diabetes and hypertension, hypercholesterolemia is an independent condition, associated with female sex and increased BMI;
- Hypercholesterolemia is present in up to 52.8% of patients, we suggest the need for a more accurate and broader NCD screening in chronic patients in Mozambique.
